# Genetic Interactions Among *Ghd7*, *Ghd8*, *OsPRR37* and *Hd1* Contribute to Large Variation in Heading Date in Rice

**DOI:** 10.1186/s12284-019-0314-x

**Published:** 2019-07-15

**Authors:** Bo Zhang, Haiyang Liu, Feixiang Qi, Zhanyi Zhang, Qiuping Li, Zhongmin Han, Yongzhong Xing

**Affiliations:** 1 National Key Laboratory of Crop Genetic Improvement and National Center of Plant Gene Research (Wuhan), Huazhong Agricultural University, Wuhan, 430070 China; 2 Hubei collaborative Innovation Center for Grain Industry, Yangtze University, Jingzhou, 434100 China

**Keywords:** Rice, Heading date, Genetic interaction, Alternative function, Genotype combination, Correlation, Spikelets per panicle

## Abstract

**Background:**

Heading date is crucial for rice reproduction and geographic expansion. Many heading date genes are sensitive to photoperiod and jointly regulate flowering time in rice. However, it is not clear how these genes coordinate rice heading.

**Results:**

Here, we performed a genetic interaction analysis among four major rice heading date genes *Ghd7*, *Ghd8*, *OsPRR37/Ghd7.1* (hereafter *PRR37*) and *Hd1* in the near-isogenic background under both natural long-day (NLD) and natural short-day (NSD) conditions. The 4-gene segregating population exhibited a large heading date variation with more than 95 days under NLD and 42 days under NSD conditions. Tetragenic, trigenic and digenic interactions among these four genes were observed under both conditions but more significant under NLD conditions. In the functional *Hd1* backgrounds, the strongest digenic interaction was *Ghd7* by *Ghd8* under NLD but was *Ghd7* by *PRR37* under NSD conditions. Interestingly, *PRR37* acted as a flowering suppressor under NLD conditions, while it functioned alternatively as an activator or a suppressor under NSD conditions depending on the status of the other three genes. Based on the performances of 16 homozygous four-gene combinations, a positive correlation between heading date and spikelets per panicle (SPP) was found under NSD conditions, but changed to a negative correlation when heading date was over 90 days under NLD conditions.

**Conclusions:**

These results demonstrate the importance of genetic interactions in the rice flowering regulatory network and will help breeders to select favorable combinations to maximize rice yield potential for different ecological areas.

**Electronic supplementary material:**

The online version of this article (10.1186/s12284-019-0314-x) contains supplementary material, which is available to authorized users.

## Background

Heading date, a crucial trait for rice expansion to high latitudes, is determined by both genetic factors and environmental cues (Andres and Coupland 2012). Cultivars with an appropriate heading date will be conductive to high grain yield by fully utilizing the light and temperature resources in their growing regions (Zhang et al. 2015a).

In the last two decades, dozens of quantitative trait loci (QTLs) for rice heading date have been cloned by using biparental populations, germplasm resources and mutants with forward- or reverse-genetics approaches (Yamamoto et al. 2012; Hori et al. 2016; Yano et al. 2016). Among these genes, several major QTLs, especially those cloned from natural variations, have pleiotropic effects on heading date, plant height and grain yield, which have been widely subjected to artificial selection in the process of rice genetic improvement. For example, *Heading date1* (*Hd1*), the homolog of *Arabidopsis CONSTANS* (*CO*), encodes a zinc finger CCT (CO, CO-LIKE and TIMING OF CAB1) domain and acts as a major flowering activator in rice (Yano et al. 2000; Zhang et al. 2017). *Hd1* delays heading date in some varieties under long-day (LD) conditions by interacting with other flowering genes such as *Ghd7*, resulting in a taller plant and more grain yield (Nemoto et al. 2016; Zhang et al. 2017). *Ghd7* is a rice-specific gene encoding a CCT domain protein and is important for heading date, grain yield, rice adaptation and drought resistance (Xue et al. 2008; Weng et al. 2014). Another major QTL, *Ghd8* (allelic to *Hd5* and *DTH8*), encodes a HAP3 subunit of heterotrimeric heme activator protein (HAP) and simultaneously controls heading date, plant height and grain number (Wei et al. 2010; Yan et al. 2011; Fujino et al. 2013). *OsPRR37*, allelic to *Ghd7.1*, *DTH7* and *Hd2* and encoding a PSEUDO-RESPONSE REGULATOR 7-like protein harboring the CCT domain, greatly represses heading and increases grain yield under LD conditions (Koo et al. 2013; Liu et al. 2013; Gao et al. 2014). Natural variations in *OsPRR37/Ghd7.1* also contribute to rice cultivation at a wide range of latitudes (Koo et al. 2013; Yan et al. 2013). It was initially demonstrated that these genes are in separate branches in the flowering regulatory network and have partially unrelated effects on transcription level (Brambilla and Fornara 2013; Song et al. 2015).

Photoperiod sensitivity largely determines heading date in rice. There are two independent genetic pathways involved in photoperiod sensitivity. One is the *OsGI-Hd1-Hd3a* pathway, which is conserved with the *GI-CO-FT* pathway in *Arabidopsis* (Shrestha et al. 2014). *Hd1* is upregulated by *OsGI* and activates the expression of *Hd3a* to promote rice heading under both short-day (SD) and LD conditions (Hayama et al. 2003; Zhang et al. 2017). Another is the *Ehd1-Hd3a* pathway, a unique pathway in rice regulated by many genes (Doi et al. 2004; Tsuji et al. 2011). Among these genes, *Ehd2*, *Ehd3*, *Ehd4* and *OsMADS51* always promote rice heading by directly or indirectly upregulating the expression of *Ehd1* under both SD and LD conditions (Kim et al. 2007; Matsubara et al. 2008; Matsubara et al. 2011; Gao et al. 2013). In contrast, other genes including *Ghd7*, *Ghd8*, *OsPRR37*, *Hd16*, *OsCOL4* and *OsCOL10* repress the expression of *Ehd1*, resulting in late flowering under LD conditions (Xue et al. 2008; Lee et al. 2010; Yan et al. 2011; Hori et al. 2013; Yan et al. 2013; Tan et al. 2016). The recent finding that the Ghd7-Hd1 complex represses *Ehd1* by binding to a cis-regulatory region in the *Ehd1* 5′-UTR suggested that *Hd1* was integrated into the rice-specific genetic pathway (Nemoto et al. 2016).

Our previous studies indicated that *Ghd7* and *Ghd8* in the ZS97 background greatly delayed heading date (non-heading) under NLD conditions because of the presence of *Hd1*, indicating a strong genetic interaction among *Ghd7*, *Ghd8* and *Hd1* (Zhang et al. 2015a). PRR37 shared the conserved CCT domain with Hd1 and Ghd7, and formed a heterotrimer with Ghd8 and NF-YCs similar to Hd1 (Zhang et al. 2015b; Goretti et al. 2017). Thus, we hypothesized that *PRR37* is involved in genetic interactions with the three other genes. To test this hypothesis, we further conducted genetic interaction analysis among *Ghd7*, *Ghd8*, *PRR37* and *Hd1* in the ZS97 background under NLD and NSD conditions in this study. Tetragenic, trigenic and digenic interactions among these four genes were observed under both conditions. *PRR37* always acts as a flowering suppressor under NLD conditions but exhibits an alternative function (either suppression or activation) in heading date under NSD conditions.

## Materials and methods

### Construction of NILs and segregating populations

We previously developed a near-isogenic line (NIL1) pyramiding functional *Ghd7*^*MH63*^ and *Ghd8*^*9311*^ in the ZS97 background (Zhang et al. 2015a). Another near-isogenic line (NIL2) in the ZS97 background, which harbored functional *PRR37*^*TQ*^ and nonfunctional *hd1*^*TQ*^ derived from Teqing (TQ), was crossed with NIL1. Therefore, NIL-F_1_ plants carried heterozygous *Ghd7*, *Ghd8*, *PRR37* and *Hd1* (Additional file 1: Figure S1a; Table S1). The NIL-F_2_ population was developed by self-crossing a NIL-F_1_ plant that was genotyped by using the RICE6K SNP array (Yu et al. 2014) (Additional file 1: Figure S1b). To avoid genetic background noise, a NIL-F_2_ individual harboring heterozygous alleles at all four of these genes was used to produce a NIL-F_3_ population by self-pollination. All individuals of the NIL-F_2_ and NIL-F_3_ populations were genotyped at these four gene loci. According to the genotypes of the NIL-F_3_ population, 8 NIL-F_3_ plants, each carrying heterozygous *PRR37* but with different homozygous combinations of the other three genes, were used to generate 8 NIL-F_4_ populations for estimating the genetic effects of *PRR37*. Sixteen NIL-F_3_ plants with different homozygous four-gene combinations were selected to generate 16 four-gene homozygous lines for evaluating yield performance.

### Field experiments and growth conditions

Rice seeds were sown in a seedling bed in the middle of May at the experimental station of Huazhong Agricultural University, Wuhan, China (30.5°N). The 25-day-old seedlings were transplanted into the field with a distance of 16.5 cm between plants within a row and 26.5 cm between rows. The plants were subsequently grown in the field under NLD conditions (a day length of more than 13.5 h) until the beginning of August (Additional file 1: Table S2). For the field experiments under NSD conditions, the plant materials were sown in Lingshui, Hainan (18.5°N), at the beginning of December and were transplanted into the field after 1 month, at the same planting density as that used in Wuhan, and grown under an average day length of less than 12.5 h from December to April (Additional file 1: Table S2).

The NIL-F_2_ population consisting of 680 individuals was grown in Wuhan in 2016. Excluding the marginal plants and abnormally growing individuals, 509 individuals were used for analysis of genetic interactions among *Ghd7*, *Ghd8*, *PRR37* and *Hd1* under NLD conditions. A total of 900 NIL-F_3_ plants derived from an F_2_ individual segregating for these four genes were grown in Lingshui from Dec 2016 to Apr 2017, and a total of 679 non-marginal individuals were used for analysis of genetic interactions among these four genes under NSD conditions. Eight NIL-F_4_ populations were grown in Wuhan (~ 60 plants per population) in summer 2017 (from May to October) and in Lingshui (~ 40 plants per population) in winter (from Dec 2017 to Apr 2018). Meanwhile, 16 four-gene homozygous lines were also grown in Wuhan and Lingshui in summer and winter of 2017, respectively. Three additional *PRR37*-segregating population (~ 80 plants per population) with the backgrounds *Ghd7Ghd8Hd1*, *Ghd7Ghd8hd1* and *Ghd7ghd8Hd1* were also grown in Lingshui in winter 2017. In addition, four plants of each four-gene homozygous combination were grown in the field to implement a short-day treatment with a day length of 11 h and darkness of 13 h in the summer of 2018. A set of plants from these genotypes were planted in the same field at the same density under NLD conditions and served as the control group.

### DNA extraction, polymerase chain reaction and genotyping

At the tillering stage, leaf blades were collected for DNA extraction using a modified cetyl-trimethyl ammonium bromide (CTAB) method (Murray and Thompson 1980). Genomic DNA was amplified using rTaq polymerase from TaKaRa in Buffer I according to the manufacturer’s indications. For each PCR reaction, DNA was initially incubated for 5 minutes at 95 °C, followed by 35 cycles of amplification (95 °C for 30 s, 58 °C for 30 s and 72 °C for 30 s). The simple sequence repeat (SSR) marker MRG4436, which is tightly linked to *Ghd7*, and the functional markers Z9M, InDel37 and S56 designed from *Ghd8*, *PRR37* and *Hd1* (Additional file 1: Table S1), respectively, were used to genotype the individuals of all populations and NILs. All markers used for genotyping are listed in (Additional file 1: Table S7).

### RNA extraction and qRT-PCR analysis

Seedlings were grown in a seedbed under NLD conditions for 30 days and were subsequently transplanted to a plot in the field for the short-day treatment (started on the 11th of June, light treatment from 7:00 am to 6:00 pm every day). After treatment for 15 days (from the 11th to 26th of June), the young leaves in the short-day treatment and control group (treated with LD condition, i.e., more than 14 h day length per day from the 11th to 26th of June) were collected at 9:00 am for RNA extraction. For each genotype, leaves from three different individuals were collected as biological replicates. Total RNA was extracted using TRIzol reagent (TransGen Biotech, Beijing) and treated with DNase I (Invitrogen, USA). cDNA was synthesized from 3 μg of RNA using SuperScript III Reverse Transcriptase (Invitrogen, USA). The quantitative analysis of gene expression was performed with SYBR Premix ExTaq reagent (TaKaRa, Dalian) on the ABI ViiA7 Real-time PCR System (Applied Biosystems, USA). The data were analyzed using the relative quantification method. The primers used for real-time PCR are listed in (Additional file 1: Table S7).

### Trait measurement and data analysis

Heading date was individually scored as the number of days from sowing to the emergence of the first panicle on the plant. The total number of spikelets per plant was measured by the Yield Traits Scorer (Yang et al. 2014). The number of spikelets per panicle (SPP) of each homozygous combination line was recorded as the total number of spikelets divided by the number of panicles. The comparison between genotypes was performed by Student’s *t*-test. To verify the existence of high order genetic interactions, the three-way ANOVA or factorial ANOVA were performed under the condition of fixation of the allele at the fourth gene. The statistical significance of three-way interactions was evaluated by a general liner model (GLM) using the program STATISTICA 8.0 (Statsoft 1995).

## Results

### Composition of major heading date genes in ZS97

Our previous studies confirmed that ZS97 carried a functional allele of *Hd1* and nonfunctional alleles of *Ghd7*, *Ghd8* and *PRR37/Ghd7.1* (Xue et al. 2008; Yan et al. 2011; Yan et al. 2013; Zhang et al. 2017). To clarify the genetic background on heading date, the coding sequences and functional nucleotide polymorphisms of other 10 major flowering genes were downloaded from the reference genome of ZS97 and Rice SNP-Seek Database, respectively (Alexandrov et al. 2015; Song et al. 2018; Wang et al. 2018). Alignment of coding sequence were used to compare allele identity between ZS97 and varieties used in previous studies (Additional file 1: Table S3). Alleles of *DTH3/OsMADS50* and *Hd6* were the same as the one carried by Dianjingyou 1 and Kasalath, respectively, which were the functional alleles (Takahashi et al. 2001; Lee et al. 2004; Bian et al. 2011). The haplotypes of *Hd16/EL1*, *Hd3a* and *Ehd1* were identified as Type 4, Type 3 and Type 6, respectively, which were also confirmed as the functional types (Takahashi et al. 2009; Hori et al. 2013; Kwon et al. 2014). Allele of *Ehd4* in ZS97 was the same as the 93–11 haplotype, Hap_2, which was a weak functional allele demonstrated by transgenic verification (Gao et al. 2013). The haplotype of *Hd17* in ZS97 was consistent with that in Koshihikari, which was a weak allele compared with Nipponbare (Matsubara et al. 2012). Allele of *Hd18* in ZS97 was the same as that in Hayamasari, acted as a weak allele (Shibaya et al. 2016). The haplotype of *DTH2* in ZS97 was consistent with Group A1, which was a nonfunctional allele (Wu et al. 2013). *RFT1* in ZS97 belonged to Type IIb with E105K variation and also exhibited a loss of function (Zhao et al. 2015).

### The genetic interactions among *Ghd7*, *Ghd8*, *PRR37* and *Hd1* under NLD conditions

**Fig. 1:**
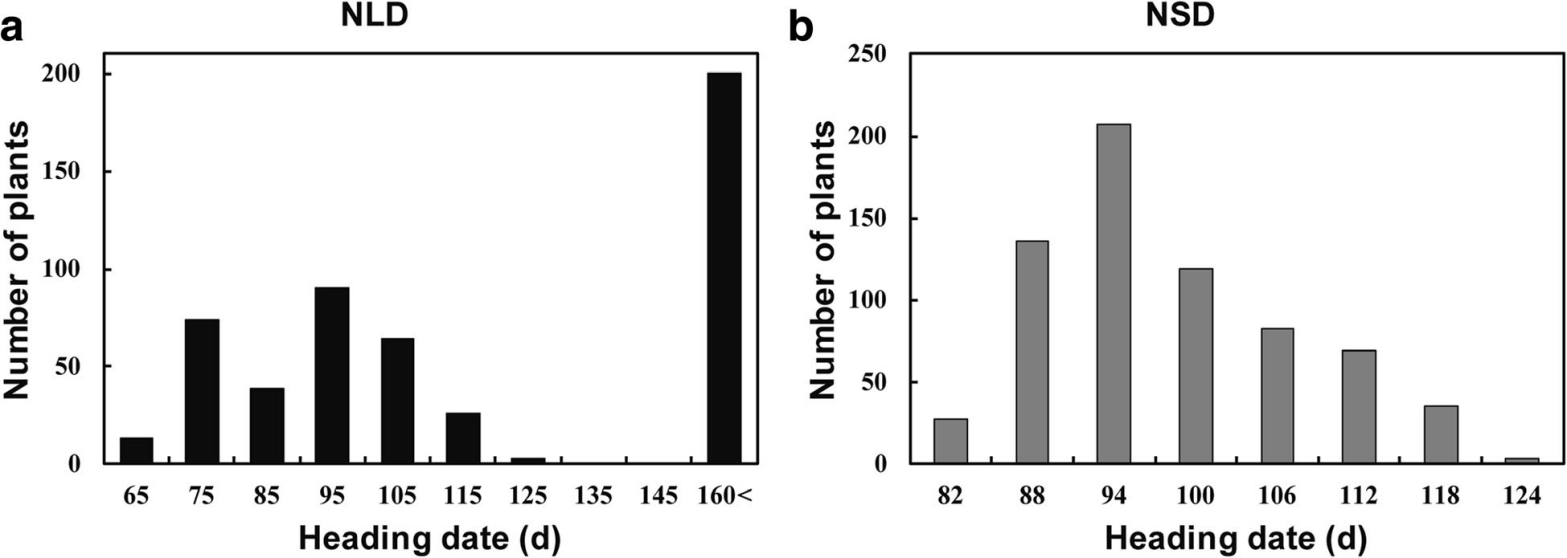
The heading date distribution of 4-gene segregating populations under NLD and NSD conditions. The heading date distribution of NIL-F_2_ population under NLD conditions (**a**) and of NIL-F_3_ population under NSD conditions (**b**). “160<”, non-heading after 160 days from sowing

The NIL-F_1_ plant carrying heterozygous alleles at these four genes (Additional file 1: Figure S1a) was genotyped by the RICE6K SNP array. More than 90% of the NIL-F_1_ plant background was consistent with ZS97, but the segments harboring *Ghd7*, *Ghd8*, *PRR37* and *Hd1* were heterozygous. The segments harboring other 10 flowering gene regions were fixed with ZS97 genotype in the NIL-F_1_ plant (Additional file 1: Figure S1b). In the NIL-F_2_ population, large variation in heading date was observed, ranging from 65 days to no heading after 160 days under NLD conditions (Fig. 1a). For convenience, 160 days was recorded as the heading date of these non-heading plants. Two-way and three-way ANOVA separately showed that all 6 pairs of digenic interactions and 4 trigenic interactions were highly significant (Additional file 1: Table S4). Four-way ANOVA revealed that the tetragenic interaction among these four genes was also highly significant (Additional file 1: Table S4). To better understand the four-way interaction, we classified the populations into three subpopulations based on *Hd1* genotypes: homozygous *Hd1*, heterozygous *Hd1* (*Hd1*^*H*^) and homozygous *hd1*. A significant three-way interaction was detected among *Ghd7*, *Ghd8* and *PRR37* at *P* < 1.0E-10 in both the *Hd1* and *Hd1*^*H*^ backgrounds and at *P* = 6.9E-04 in the *hd1* background (Additional file 1: Figure S2a-c; Table 1). Additionally, all digenic interactions were detected among *Ghd7*, *Ghd8* and *PRR37*. The *Ghd7* by *Ghd8* interaction contributed more to heading date variation than the other digenic interactions. The square of this interaction accounted for 5.9% and 5.8% of the total sum-of-squares in the *Hd1* and *Hd1*^*H*^ backgrounds, respectively, and 5.8% of that in the nonfunctional *hd1* background (Table 1). The main effects of *Ghd7*, *Ghd8* and their digenic interaction effects explained more than 70% of the variation in heading date in both the *Hd1* and *Hd1*^*H*^ backgrounds. The genetic square of *PRR37* accounted for 17.0% of the total sum-of-squares in the *hd1* background, which was much larger than that observed in the *Hd1* and *Hd1*^*H*^ backgrounds (Table 1). Taken together, these results revealed that a strong trigenic interaction existed among *Ghd7*, *Ghd8* and *PRR37* regardless of the genotype of *Hd1*, and the interaction between *Ghd7* and *Ghd8* showed the strongest digenic interaction among these three genes under NLD conditions.

**Table 1:** Three-way ANOVA analysis of *Ghd7*, *Ghd8* and *PRR37* in NIL-F_2_ population under NLD conditions

Effect	D	*hd1* (*n* = 138; ^a^74d-128d)			*Hd1*^*H*^ (*n* = 242; 67d-^b^160d)			*Hd1* (*n* = 129; 64d-160d)		
		F	*P*	G:T (%)	F	*P*	G:T (%)	F	*P*	G:T (%)
*Ghd7*	2	787.7	< 1.0E-10	28.6	12145.5	< 1.0E-10	29.6	9627.4	< 1.0E-10	31.9
*Ghd8*	2	800.5	< 1.0E-10	29.1	14400.5	< 1.0E-10	35.1	11278.0	< 1.0E-10	37.4
*PRR37*	2	468.3	< 1.0E-10	17.0	747.6	< 1.0E-10	1.8	799.1	< 1.0E-10	2.6
*Ghd7 by Ghd8*	4	80.1	< 1.0E-10	5.8	1183.4	< 1.0E-10	5.8	893.3	< 1.0E-10	5.9
*Ghd7 by PRR37*	4	9.6	1.1E-06	0.7	116.5	< 1.0E-10	0.6	239.9	< 1.0E-10	1.6
*Ghd8 by PRR37*	4	21.1	< 1.0E-10	1.5	34.8	< 1.0E-10	0.2	31.1	< 1.0E-10	0.2
*Ghd7 by Ghd8 by PRR37*	8	3.7	6.9E-04	0.5	88.5	< 1.0E-10	0.9	63.8	< 1.0E-10	0.8

^a^Range of heading date variation, ^b^No heading but recorded as 160 days; *Hd1*^*H*^ heterozygous allele of *Hd1*, *DF* Degree of freedom, *G:T* Ratio of the genetic to the total of sum-of-squares

### The genetic interactions among *Ghd7*, *Ghd8*, *PRR37* and *Hd1* under NSD conditions

The heading date variation of NIL-F_3_ population exhibited a continuous distribution ranging from 82 days to 124 days (Fig. 1b). Accordingly, all digenic and trigenic interactions (except the *Ghd8* by *PRR37* by *Hd1* interaction) among these four genes were significant under NSD conditions (Additional file 1: Table S4). A significant tetragenic interaction was also observed in the NIL-F_3_ (Additional file 1: Table S4). Following the analysis performed for NLD conditions, this population were also classified into 3 classes according to *Hd1* genotypes. Significant interactions were identified among *Ghd7*, *Ghd8* and *PRR37* in the *hd1*, *Hd1*^*H*^ and *Hd1* backgrounds (Additional file 1: Figure S2d-f; Table 2). However, the digenic interactions among these three genes were different from those detected under NLD conditions. The *Ghd7* by *PRR37* interaction contributed much more to heading date variation than the other two digenic interactions in the functional *Hd1* backgrounds, in which the genetic square accounted for 20.3% and 20.4% of the total sum-of-squares in the *Hd1* and *Hd1*^*H*^ backgrounds, respectively (Table 2). Notably, the effect of *Ghd7* on heading date was the strongest under NSD conditions, explaining 58%, 21.7% and 29.1% of the variation in the *hd1*, *Hd1*^*H*^ and *Hd1* backgrounds, respectively (Table 2). These results indicated that *Ghd7*, *Ghd8* and *PRR37* interacted under NSD conditions and the *Ghd7* by *PRR37* interaction showed the strongest epistatic effect among the digenic interactions in the functional *Hd1* backgrounds.

**Table 2:** Three-way ANOVA analysis of *Ghd7*, *Ghd8* and *PRR37* in NIL-F_3_ population under NSD conditions

Effect	D	*hd1*(*n* = 165; ^a^96d-127d)			*Hd1*^*H*^ (*n* = 339; 86d-118d)			*Hd1*(*n* = 175; 82d-122d)		
		F	*P*	G:T (%)	F	*P*	G:T (%)	F	*P*	G:T (%)
*Ghd7*	2	371.1	< 1.0E-10	58.0	144.5	< 1.0E-10	21.7	107.0	< 1.0E-10	29.1
*Ghd8*	2	11.2	3.2E-05	1.7	8.4	2.9E-04	1.3	36.2	< 1.0E-10	9.8
*PRR37*	2	16.9	2.6E-07	2.6	55.7	< 1.0E-10	8.4	10.9	4.0E-05	2.9
*Ghd7 by Ghd8*	4	23.1	< 1.0E-10	7.2	9.6	2.4E-07	2.9	14.5	5.4E-10	7.9
*Ghd7 by PRR37*	4	13.0	5.0E-09	4.1	67.9	< 1.0E-10	20.4	37.4	< 1.0E-10	20.3
*Ghd8 by PRR37*	4	15.0	3.1E-10	4.7	19.5	< 1.0E-10	5.9	6.5	7.6E-05	3.5
*Ghd7 by Ghd8 by PRR37*	8	3.0	4.1E-03	1.9	8.3	3.1E-10	5.0	4.4	9.3E-05	4.8

^a^Range of heading date variation; *Hd1*^*H*^ Heterozygous allele of *Hd1*, *DF* Degree of freedom, *G:T* Ratio of the genetic to the total of sum-of-squares

### *PRR37* acts as a heading date suppressor under NLD conditions

**Fig. 2:**
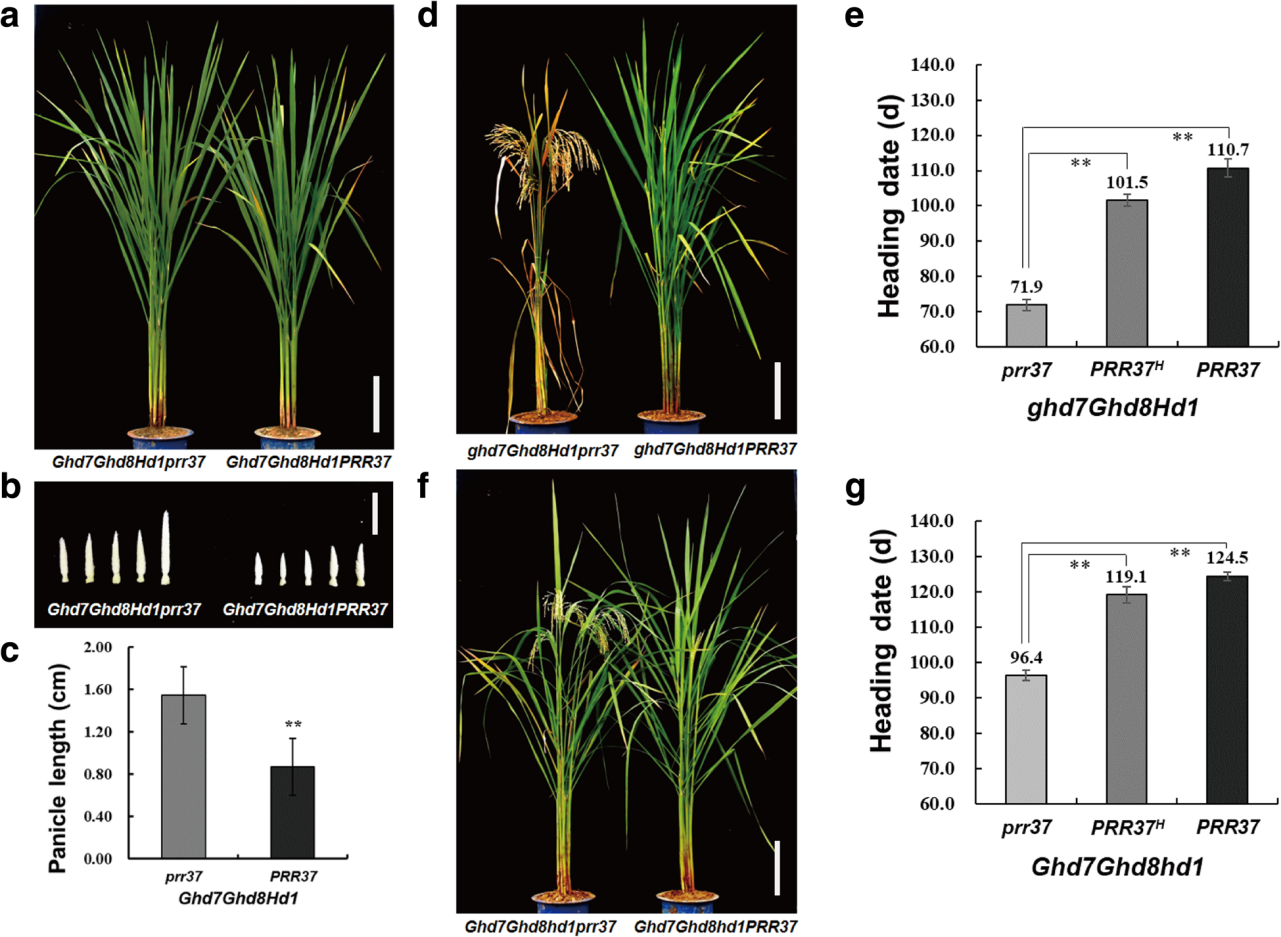
*PRR37* delays heading date under NLD conditions. Non-heading plants of *prr37* and *PRR37* **a** in the *Ghd7Ghd8Hd1* background, **b** their young panicles of main stems and **c** the comparison of panicle length. **d** The plants of *ghd7Ghd8Hd1PRR37* and *ghd7Ghd8Hd1prr37*. **e** The large effect of *PRR37* on heading date in the *ghd7Ghd8Hd1* background. **f** The plants of *Ghd7Ghd8hd1PRR37* and *Ghd7Ghd8hd1prr37*. **g** The strong effect of *PRR37* on heading date in the *ghd7Ghd8Hd1* background. **, *P* < 0.01 based on Student’s *t*-test; *n* = 15 for each combination in **c**, and *n* ≥ 10 for each genotype in **e** and **g**. *PRR37*^*H*^, the heterozygous allele of *PRR37*. Scale bars: 20 cm for **a**, **d** and **f**, and 1 cm for **b**

To estimate the additive and dominance effects of *PRR37* in different genetic backgrounds under NLD conditions, we developed 8 *PRR37*-segregating populations (NIL-F_4_) with different homozygous combinations of the other three genes. The NIL-F_4_ population with the *Ghd7Ghd8Hd1* background did not head even after October 24th, when the low temperature is unfavorable to rice growing in Wuhan (Fig. 2a). Therefore, no data were used to evaluate the genetic effect of *PRR37* in this population (Table 3). We merged the 8 NIL-F_4_ populations for interaction analysis because these populations shared similar genetic background and were grown in the same condition. All digenic and trigenic interactions or even tetragenic interaction among these four genes were also highly significant (Additional file 1: Figure S3a-c; Table S5). To confirm whether *PRR37* also delayed rice heading in the *Ghd7Ghd8Hd1* background, we took the young panicles of the main stems of the two homozygous combinations, namely, *Ghd7Ghd8Hd1PRR37* and *Ghd7Ghd8Hd1prr37*, on September 30th and compared their lengths (Fig. 2b). The young panicle length of *Ghd7Ghd8Hd1PRR37* (0.87 cm) was significantly shorter than that of *Ghd7Ghd8Hd1prr37* (1.55 cm), which suggested that *PRR37* suppressed heading in the *Ghd7Ghd8Hd1* background (Fig. 2c). The additive effect of *PRR37* in the other 7 populations ranged from 5.6–19.4 days, indicating that *PRR37* always plays as a suppressor of heading date in these backgrounds under NLD conditions (Table 3). The dominance effects and degrees of dominance of *PRR37* ranged from 2.4–10.7 days and from 0.28–0.93, respectively (Table 3). Accordingly, we observed large heading date variations in the *ghd7Ghd8Hd1* and *Ghd7Ghd8hd1* backgrounds, ranging from 69 to 115 days and from 94 to 127 days, respectively (Table 3). The effects of *PRR37* on heading date were 38.8 and 28.1 days in these two backgrounds, respectively, which were much larger than that in the *ghd7ghd8hd1* background (Fig. 2d-g; Table 3). These results revealed that the large genetic effects of *PRR37* on heading date were dependent on the combinations of *Ghd7*, *Ghd8* and *Hd1*.

**Table 3:** The genetic effects of *PRR37* on heading date in 8 NIL-F_4_ populations under NLD conditions

Background	Size	Heading date (d)						
		Range	*prr37*	*PRR37* ^*H*^	*PRR37*	A	D	|D/A|
*ghd7ghd8hd1*	47	70–88	71.0 ± 1.7	81.3 ± 2.3	84.7 ± 1.8	6.8	3.4	0.50
*ghd7Ghd8hd1*	51	74–97	75.7 ± 1.2	84.9 ± 1.8	92.7 ± 2.3	8.5	NS	
*Ghd7ghd8hd1*	52	77–93	79.1 ± 1.8	87.0 ± 1.6	90.2 ± 1.8	5.6	2.4	0.43
*Ghd7Ghd8hd1*	59	94–127	96.4 ± 1.4	119.1 ± 2.4	124.5 ± 1.2	14.0	8.7	0.62
*ghd7ghd8Hd1*	47	59–82	61.5 ± 1.5	73.2 ± 2.7	79.8 ± 1.7	9.1	2.5	0.28
*ghd7Ghd8Hd1*	47	69–115	71.9 ± 1.5	101.5 ± 1.7	110.7 ± 2.5	19.4	10.2	0.53
*Ghd7ghd8Hd1*	58	80–108	81.6 ± 1.3	103.7 ± 1.3	104.5 ± 1.6	11.5	10.7	0.93
*Ghd7Ghd8Hd1*	60	NH	NH	NH	NH			

Size, the number of plants of segregating population; *PRR37*^*H*^, heterozygous allele of *PRR37*; A, additive effect; D, dominance effect; |D/A|, the degree of dominance; NS, no significance; NH, no heading

## Alternative functions of *PRR37* in repressing or promoting heading under NSD conditions

**Fig. 3:**
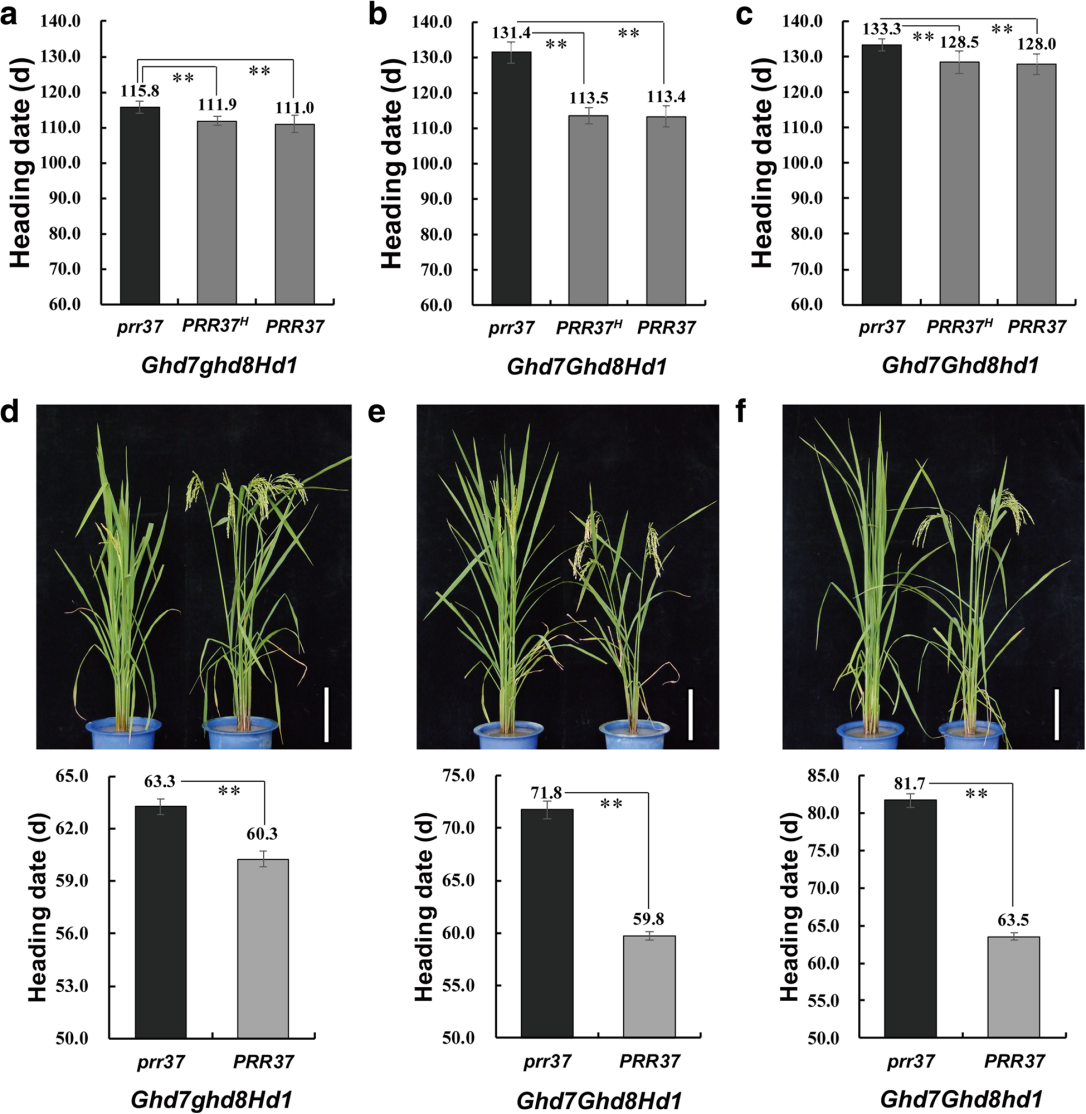
*PRR37* promotes rice heading in the specific backgrounds under NSD and SD conditions. Comparisons of heading date among different alleles of *PRR37* in the backgrounds *Ghd7ghd8Hd1* **a**, *Ghd7Ghd8Hd1* **b** and *Ghd7Ghd8hd1* **c** under NSD conditions (*n* ≥ 10 for each combination). **d**-**f**, Pictures (top) and heading dates (bottom) of *prr37* and *PRR37* in each corresponding background under SD conditions (*n* = 4 for each combination). **, *P* < 0.01 based on Student’s *t*-test. *PRR37*^*H*^, the heterozygous allele of *PRR37*. Scale bars: 20 cm for **d**, **e** and **f**

Heading dates of these 8 *PRR37*-segregating populations (NIL-F_4_) also exhibited a continuous distribution ranging from 95 to 135 days under NSD conditions (Additional file 1: Figure S3d). We merged these 8 populations together for interaction analysis. Accordingly, most of digenic and trigenic interactions and tetragenic interaction among these four genes were significant (Additional file 1: Figure S3e-f; Table S5). The additive effects of *PRR37* were 1.8 days, 5.0 days and 2.2 days in the *ghd7ghd8hd1*, *ghd7ghd8Hd1* and *ghd7Ghd8Hd1* backgrounds, respectively (Table 4), indicating that *PRR37* acted as a flowering suppressor in these three backgrounds. However, the effect on delaying heading date was much smaller than that observed under NLD conditions. The genetic effect of *PRR37* disappeared in the *ghd7Ghd8hd1* and *Ghd7ghd8hd1* backgrounds. Interestingly, a converse effect of *PRR37* on heading date was observed in the *Ghd7ghd8Hd1*, *Ghd7Ghd8Hd1* and *Ghd7Ghd8hd1* backgrounds comparing with that observed under NLD conditions. The additive effects of *PRR37* in these three backgrounds were − 2.0 days, − 9.9 days and − 3.7 days, respectively (Table 4). Therefore, it seemed that *PRR37* acted as a heading activator in these three backgrounds. Three additional *PRR37*-segregating populations with the *Ghd7ghd8Hd1*, *Ghd7Ghd8Hd1* and *Ghd7Ghd8hd1* backgrounds were used to verify this finding. Compared to *prr37*, *PRR37* promoted rice heading by 4.8 days, 18.0 days and 5.3 days in these three backgrounds, respectively (Fig. 3a-c). In addition, *PRR37* promoted heading by 3.0 days, 12.0 days and 18.2 days in these three backgrounds in the 11-h light treatment, respectively (Fig. 3d-f). These data clearly demonstrated that *PRR37*acted as a heading activator in the *Ghd7ghd8Hd1*, *Ghd7Ghd8Hd1* and *Ghd7Ghd8hd1* backgrounds under NSD conditions. The dominance effects and degrees of dominance of *PRR37* in these 8 populations ranged from − 7.3 to 1.9 days and from 0.38 to 0.88, respectively (Table 4), suggesting that the genetic effects of *PRR37* were largely influenced by the genetic background.

**Table 4:** The genetic effects of *PRR37* on heading date in 8 NIL-F_4_ populations under NSD conditions

Background	Size	Heading date (d)						
		Range	*prr37*	*PRR37* ^*H*^	*PRR37*	A	D	|D/A|
*ghd7ghd8hd1*	40	112–122	114.9 ± 1.5	118.2 ± 1.5	118.4 ± 2.0	1.8	1.6	0.88
*ghd7Ghd8hd1*	39	110–117	114.9 ± 1.6	114.7 ± 1.0	113.5 ± 1.6	NS	NS	
*Ghd7ghd8hd1*	38	120–126	123.2 ± 0.8	123.3 ± 1.9	123.7 ± 1.0	NS	NS	
*Ghd7Ghd8hd1*	40	123–135	133.4 ± 1.3	127.1 ± 1.5	126.0 ± 1.2	−3.7	−2.6	0.71
*ghd7ghd8Hd1*	39	97–112	100.0 ± 2.1	106.9 ± 3.0	110.0 ± 1.4	5.0	1.9	0.38
*ghd7Ghd8Hd1*	40	102–112	104.5 ± 1.9	108.1 ± 1.7	108.9 ± 1.6	2.2	1.4	0.64
*Ghd7ghd8Hd1*	38	108–116	113.3 ± 1.6	111.9 ± 1.5	109.3 ± 1.3	−2.0	NS	
*Ghd7Ghd8Hd1*	40	113–136	133.4 ± 1.7	116.2 ± 2.3	113.6 ± 1.1	−9.9	−7.3	0.74

Size, the number of plants of segregating population; *PRR37*^*H*^, heterozygous allele of *PRR37*; Negative value indicates the functional allele of *PRR37* promotes rice heading. A, additive effect; D, dominance effect; |D/A|, the degree of dominance; NS, no significance

### Transcriptional analysis of *Ehd1* and *Hd3a* in the *Ghd7ghd8Hd1*, *Ghd7Ghd8Hd1* and *Ghd7Ghd8hd1* backgrounds

**Fig. 4:**
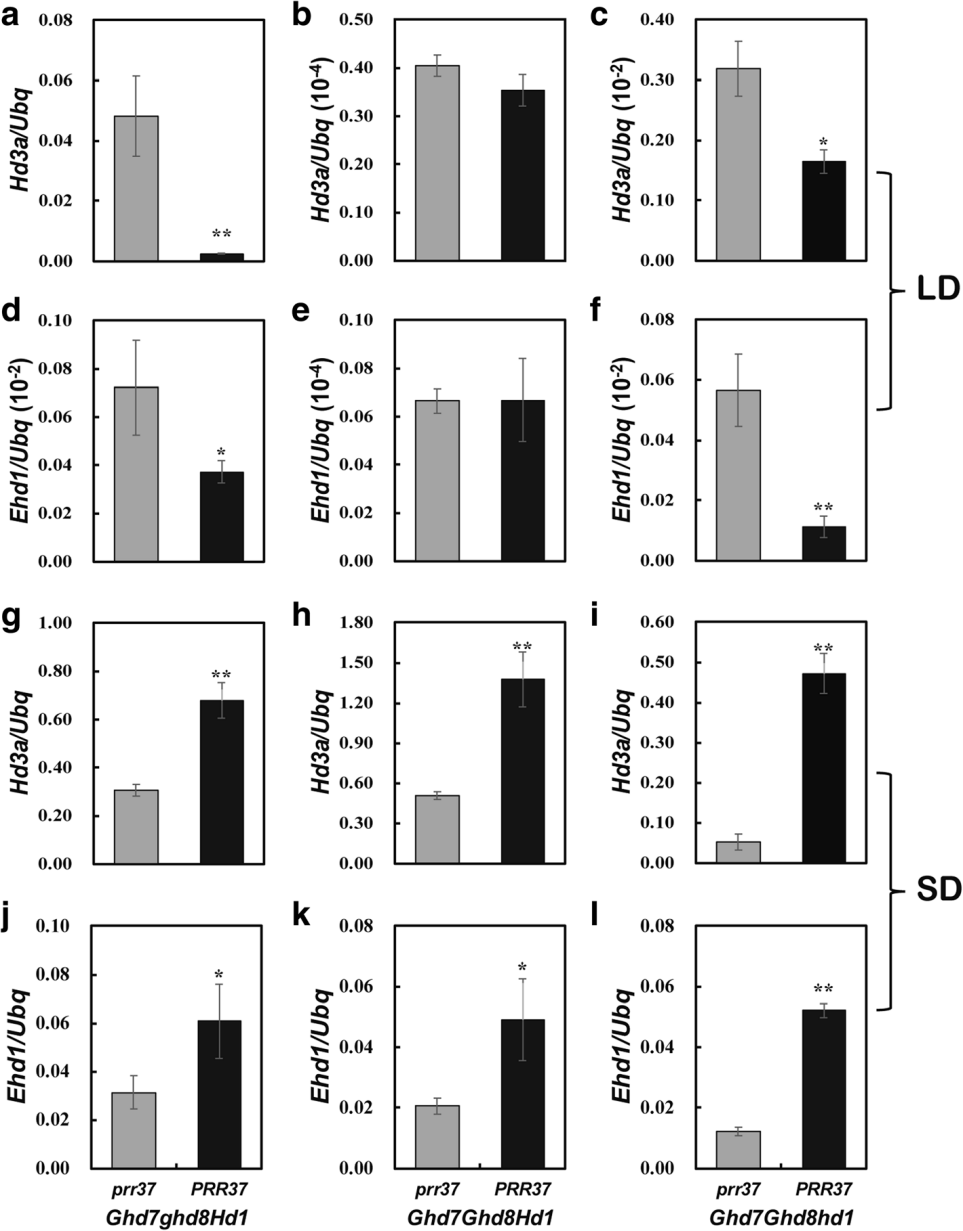
Transcript levels of *Hd3a* and *Ehd1* between *prr37* and *PRR37* under LD and SD conditions. Relative expression levels of *Hd3a* and *Ehd1* between *prr37* and *PRR37* in the *Ghd7ghd8Hd1*(**a**, **d**), *Ghd7Ghd8Hd1* (**b**, **e**) and *Ghd7Ghd8hd1* (**c**, **f**) backgrounds under LD conditions, respectively; Relative expression levels of *Hd3a* and *Ehd1* between *prr37* and *PRR37* in the *Ghd7ghd8Hd1*(**g**, **j**), *Ghd7Ghd8Hd1* (**h**, **k**) and *Ghd7Ghd8hd1* (**i**, **l**) backgrounds under SD conditions, respectively. * and **, *P* < 0.05 and *P* < 0.01 based on Student’s *t*-test, respectively

Considering that *PRR37* has an alternative function in these three backgrounds under different day-length conditions, the expression of *PRR37* downstream genes, *Ehd1* and *Hd3a*, was compared between *prr37* and *PRR37* in these three backgrounds under LD and SD conditions, respectively. The relative expression levels of *Ehd1* and *Hd3a* in *Ghd7ghd8Hd1PRR37* and *Ghd7Ghd8hd1PRR37* genotypes decreased under LD conditions but increased under SD conditions compared with those in *Ghd7ghd8Hd1prr37* and *Ghd7Ghd8hd1prr37*, respectively (Fig. 4). The expression of *Ehd1* and *Hd3a* showed no significant difference between *prr37* and *PRR37* in the *Ghd7Ghd8Hd1* background under LD conditions but increased with the presence of *PRR37* under SD conditions (Fig. 4). These results indicated that *PRR37* promoted the expression of *Ehd1* and *Hd3a* in these three backgrounds under SD conditions, resulting in an early heading date. In contrast, *PRR37* delayed rice heading in the *ghd7ghd8Hd1* background by repressing the expression of *Ehd1* and *Hd3a* under both LD and SD conditions (Additional file 1: Figure S4).

### Correlation between heading date and SPP under NLD and NSD conditions

**Fig. 5:**
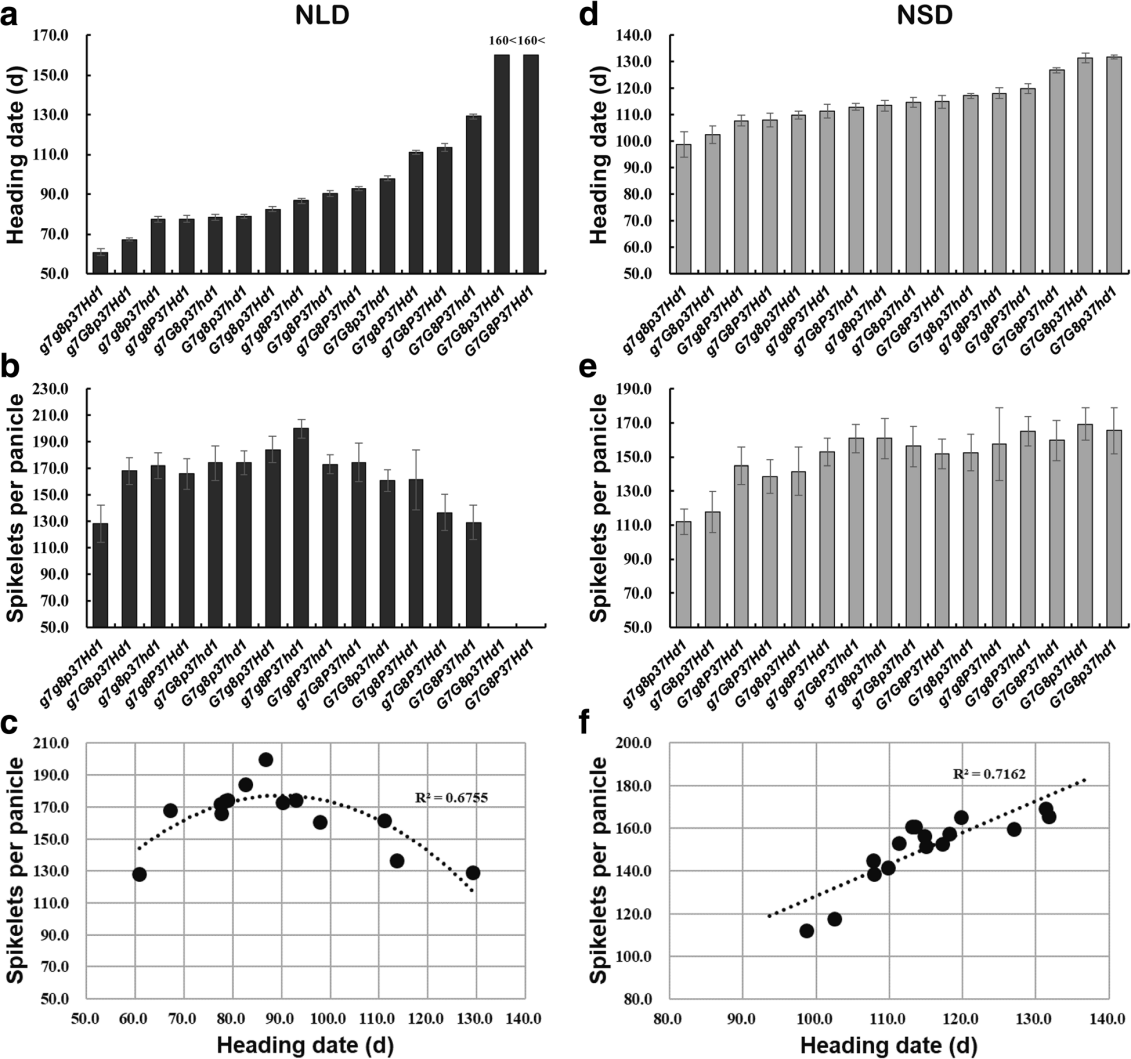
Performances of 16 4-gene homozygous combinations on heading date, SPP under NLD and NSD conditions. Heading date (**a**, **d**), SPP (**b**, **e**) and curve-fitting plots of heading date with SPP (**c, f**) under NLD and NSD conditions, respectively. The combinations in **a**, **b**, **d** and **e** are ordered by the increasing heading date. Curves fitting the trait change in **c** and **f** are calculated by the quadratic and liner equation with *R*^2^ values, respectively. *G7*, *G8*, *P37* indicate functional alleles of *Ghd7*, *Ghd8* and *PRR37*, respectively. *g7*, *g8*, *p37* indicate nonfunctional alleles of *Ghd7*, *Ghd8* and *PRR37*, respectively. “160<”, non-heading after 160 days from sowing. 20 ≤ *n* ≤ 24 for each combination under NLD conditions and 10 ≤ *n* ≤ 16 for each combination under NSD conditions

We identified the relationship between heading date and SPP on the basis of performance of 16 homozygous 4-gene combinations (Fig. 5; Additional file 1: Table S6). Under NLD conditions, the heading date of these 16 combinations exhibited a continuous distribution ranging from 60 days to 130 days except for the two non-heading combinations *Ghd7Ghd8PRR37Hd1* and *Ghd7Ghd8prr37Hd1*. The earliest heading combination was *ghd7ghd8prr37Hd1* with 60.8 days, which was the ZS97 genotype (Fig. 5a; Additional file 1: Table S6). Unexpectedly, SPP of these 14 combinations showed an inverse correlation with heading date. The SPP increased with later heading dates when the heading date was earlier than 90 days, while the SPP decreased with later heading dates when heading date was after 90 days (Fig. 5b). The curve-fitting plots of heading date with SPP under NLD conditions also revealed the inverse correlation with an inflection point at 90.0 days (Fig. 5c). The combination *ghd7ghd8PRR37hd1* had the most SPP, with 199.9 ± 7.3 under NLD conditions, and the second most was *Ghd7ghd8prr37Hd1*, with 184.1 ± 9.8 (Additional file 1: Table S6). Under NSD conditions, the heading date of the 16 combinations also showed a continuous distribution with a range from 98 days to 132 days. The combination with the earliest heading date was also the ZS97 genotype, *ghd7ghd8prr37Hd1*, at 98.7 days, while the combination with the latest heading date was *Ghd7Ghd8prr37hd1*, at 131.8 days (Fig. 5d; Additional file 1: Table S6). The SPP of these 16 combinations increased with the later heading dates, indicating that SPP was positively correlated with heading date under NSD conditions (Fig. 5e-f).

## Discussion

*Ghd7*, *Ghd8*, *PRR37/Ghd7.1* and *Hd1* are all photoperiod sensitive genes that respond to day-length changes and play important roles in rice adaptation to high latitude regions (Yano et al. 2000; Xue et al. 2008; Yan et al. 2011; Liu et al. 2013; Koo et al. 2013). Their combinations also largely determine the adaptation and yield potential of rice cultivars. Loss-of-function allele combination (NNN) and pre-existing strong allele combination (SSF) of *Ghd7*, *Ghd8* and *Hd1* allow rice cultivars to adapt to temperate and tropical regions, respectively (Zhang et al. 2015a). Loss-of-function alleles of *Ghd7*, *PRR37/DTH7* and *Hd1* contributed to early rice heading dates in the northern regions of northeast China, while functional alleles delayed heading in the southern regions of northeast China, indicating that divergent alleles of these three genes largely determined rice adaptation in northeast China (Ye et al. 2018). In this study, the combinations of *Ghd7*, *Ghd8*, *PRR37* and *Hd1* in ZS97 background exhibited stronger photoperiod sensitivity under NLD conditions than under NSD conditions. Significant digenic, trigenic or even tetragenic interactions of these four genes were detected under both conditions (Additional file 1: Table S4), but the significance detected under NLD conditions was much greater than that detected under NSD conditions, where the effects of *Ghd7*, *Ghd8* and *PRR37* were decreased. The OsHAPL1-DTH8-Hd1 complex acts as a transcriptional regulator of heading date by interacting with the HAP complex and GTFs (Zhu et al. 2017). *Ghd8/DTH8* encodes a HAP3 subunit, which can form a multicomplex with HAP2 and HAP5 (Thirumurugan et al. 2008). *Ghd7*, *PRR37* and *Hd1* encode transcription factors containing CCT domains, which are similar to HAP2 and responsible for DNA binding and protein-protein interaction (Wenkel et al. 2006; Thirumurugan et al. 2008). Thus, interactions among these genes probably indicate physical interactions among their encoding proteins or between proteins (transcriptional factors) and DNA elements (gene promoters). In addition, only strong functional and nonfunctional alleles were taken into consideration in this study. The heading date of these 16 four-gene combinations showed a continuous distribution with a range of 60–130 days and no heading under NLD conditions in Wuhan and a range of 98–132 days under NSD conditions in Hainan (Fig. 5). In nature, there are more diverse alleles for each gene (Koo et al. 2013; Zhang et al. 2015a). It is expected that different gene combinations will have similar heading dates due to the comprehensive effect of single gene and interaction effects. A better understanding of these four major flowering genes will aid in breeding design for developing cultivars for local rice production. It is noticed that these findings are derived from typical *Xian* (indica) cultivar, ZS97. It is not clear whether similar results would be obtained in *Geng* (japonica), which is worth testing in the future.

Grain yield is positively correlated with heading date, especially in low latitude areas where the temperature is warm year-round (Gao et al. 2014; Li et al. 2018). In this study, due to continuously high temperature stress during the rice flowering stage in Wuhan, the seed setting rates were significantly decreased; therefore, we analyzed the relationship between heading date and SPP instead of that between heading date and grain yield. The SPP is consistently and positively correlated with heading date under NSD conditions. Nevertheless, The SPP exhibited an inverse correlation with heading date under NLD conditions. The SPP increased with increasing days from sowing to heading when the heading date was earlier than 90 days, while it decreased with increasing days when the heading date was later than 90 days. Based on this finding, optimized combinations can be suggested for local regions to maximize rice production in indica varieties. For example, varieties with the *Ghd7Ghd8prr37Hd1* and *Ghd7Ghd8prr37hd1* combinations will produce more grains in low latitude regions (tropical regions) with short-day and warm conditions such as Hainan. In subtropical regions like Wuhan, the *ghd7ghd8PRR37hd1* and *Ghd7ghd8prr37Hd1* combinations will have the highest yield potential. In this study, the set of materials was grown at only two locations. If they were tested in multiple diverse ecological areas, the favorable gene combinations could be defined for each area.

Previous studies showed that *PRR37* inhibited heading date under LD conditions but seemed to have no effect under SD conditions (Koo et al. 2013; Liu et al. 2013; Gao et al. 2014). However, in this study, *PRR37* delayed rice heading in the *ghd7ghd8hd1*, *ghd7ghd8Hd1* and *ghd7Ghd8Hd1* backgrounds but significantly promoted heading in the *Ghd7Ghd8hd1*, *Ghd7Ghd8Hd1* and *Ghd7ghd8Hd1* backgrounds under NSD conditions (Fig. 3; Table 4), which clearly demonstrated that *PRR37* had alternative functions under SD conditions. *PRR37* suppressed heading date by inhibiting the expression of its downstream genes *Ehd1* and *Hd3a* under LD conditions. In contrast, *PRR37* acted as an activator of rice heading by promoting *Ehd1* and *Hd3a* expression in the *Ghd7Ghd8hd1*, *Ghd7Ghd8Hd1* and *Ghd7ghd8Hd1* backgrounds under SD conditions (Fig. 4). All these three backgrounds had functional allele of *Ghd7*, indicating that *Ghd7* played an essential role in the function inversion of *PRR37*. However, Ghd7 and PRR37 are both transcriptional suppressors (Weng et al. 2014; Liu et al. 2018). The effect of *Ghd7* on heading date was the largest in the 3-gene segregating populations with fixed *Hd1* genotypes, and the *Ghd7* by *PRR37* interaction was the strongest digenic interaction in these populations under NSD conditions (Table 2). Consequently, the enhanced genetic interaction between *Ghd7* and *PRR37* under SD conditions most likely attenuated the interaction of *Ghd7* with other genes, and ultimately weakened the ability of *Ghd7* and *PRR37* or their complexes to inhibit the expression of downstream genes, *Ehd1* and *Hd3a*, resulting in an early heading date. This hypothesis deserved to be further validated and improved by more genetic and molecular biology evidences.

## Conclusions

Multi-order genetic interactions among *Ghd7*, *Ghd8*, *PRR37* and *Hd1* were observed in the 4-gene segregating population under both NLD and NSD conditions. These four genes jointly determined a large heading date variation and their homozygous combinations exhibited a continuous distribution under both conditions except two non-heading combinations under NLD conditions. Coupled with the correlation between heading date and SPP, the favorable combinations were suggested for local regions to maximize rice production. Furthermore, we revealed that *PRR37* acted as a heading date suppressor under NLD conditions but it functioned alternatively under NSD conditions depending on the status of the other three genes, indicating different interactions among these four genes under different conditions. These findings revealed the importance of genetic interactions of these four genes in the photoperiod flowering pathways and contributed to a comprehensive insight into how these genes coordinate rice heading date under different day-length conditions.

## Additional file

Additional file 1: **Figure S1.** Development and genome composition of the rice populations. **Figure S2.** Genetic interaction analysis among *Ghd7*, *Ghd8*, *PRR37* and *Hd1* in the 4-gene segregating populations under NLD and NSD conditions. **Figure S3.** Genetic interaction analysis of *Ghd7*, *Ghd8*, *PRR37* and *Hd1* in the merged *PRR37*-segregating populations (NIL-F_4_) under NLD and NSD conditions. **Figure S4.** *PRR37* delays the heading date in the *ghd7ghd8Hd1* background under both LD and SD conditions. **Table S1.** Characteristics of four heading date genes and linked markers. **Table S2.** The monthly average day length of growing seasons at Wuhan and Lingshui. **Table S3.** Haplotypes of 10 heading date genes in ZS97. **Table S4.** The genetic interactions in the 4-gene segregating populations under NLD and NSD conditions. **Table S5.** The genetic interactions among four genes on the basis of the merged *PRR37*-segragating populations (NIL-F_4_) under NLD and NSD conditions. **Table S6.** The heading date and spikelets per panicle of 16 homozygous 4-gene combinations under NLD and NSD conditions. **Table S7.** Primers used in this study. (DOCX 1337 kb)

## Data Availability

The datasets supporting the conclusions of this article are included within the article and its additional files.

## Abbreviations

CCT: CO, CO-LIKE and TIMING OF CAB1
HAP: Heterotrimeric heme activator protein
LD: Long-day
NLD: Natural long-day
NSD: Natural short-day
QTLs: Quantitative trait loci
SD: Short-day
SPP: Spikelets per panicle
SSR: Simple sequence repeat
