# Supplementary material for: Genetic Interactions Among Ghd7, Ghd8, OsPRR37 and Hd1 Contribute to Large Variation in Heading Date in Rice
Source: Rice (N Y). 2019 Jul 15;12:48. doi: 10.1186/s12284-019-0314-x (PMC6629743; doi:10.1186/s12284-019-0314-x)
Supplement: Supplementary file 1 — Figure S1. Development and genome composition of the rice populations. Figure S2. Genetic interaction analysis among Ghd7, Ghd8, PRR37 and Hd1 in the 4-gene segregating populations under NLD and NSD conditions. Figure S3. Genetic interaction analysis of Ghd7, Ghd8, PRR37 and Hd1 in the merged PRR37-segregating populations (NIL-F4) under NLD and NSD conditions. Figure S4. PRR37 delays the heading date in the ghd7ghd8Hd1 background under both LD and SD conditions. Table S1. Characteristics of four heading date genes and linked markers. Table S2. The monthly average day length of growing seasons at Wuhan and Lingshui. Table S3. Haplotypes of 10 heading date genes in ZS97. Table S4. The genetic interactions in the 4-gene segregating populations under NLD and NSD conditions. Table S5. The genetic interactions among four genes on the basis of the merged PRR37-segragating populations (NIL-F4) under NLD and NSD conditions. Table S6. The heading date and spikelets per panicle of 16 homozygous 4-gene combinations under NLD and NSD conditions. Table S7. Primers used in this study. (DOCX 1337 kb) [file 12284_2019_314_MOESM1_ESM.docx]

**Supplemental material for “Genetic interactions among *Ghd7*, *Ghd8*, *OsPRR37* and *Hd1* contribute to large variation in heading date in rice**”

**Figure S1.** Development and genome composition of the rice populations.

**Figure S2.** Genetic interaction analysis among *Ghd7*, *Ghd8*, *PRR37* and *Hd1* in the 4-gene segregating populations under NLD and NSD conditions.

**Figure S3.** Genetic interaction analysis of *Ghd7*, *Ghd8*, *PRR37* and *Hd1* in the merged *PRR37*-segregating populations (NIL-F_4_) under NLD and NSD conditions.

**Figure S4**. *PRR37* delays the heading date in the *ghd7ghd8Hd1* background under both LD and SD conditions.

**Table S1.** Characteristics of four heading date genes and linked markers.

**Table S2.** The monthly average day length of growing seasons at Wuhan and Lingshui.

**Table S3.** Haplotypes of 10 heading date genes in ZS97.

**Table S4.** The genetic interactions in the 4-gene segregating populations under NLD and NSD conditions.

**Table S5.** The genetic interactions among four genes on the basis of the merged *PRR37*-segragating populations (NIL-F_4_)under NLD and NSD conditions.

**Table S6.** The heading date and spikelets per panicle of 16 homozygous 4-gene combinations under NLD and NSD conditions.

**Table S7.** Primers used in this study.


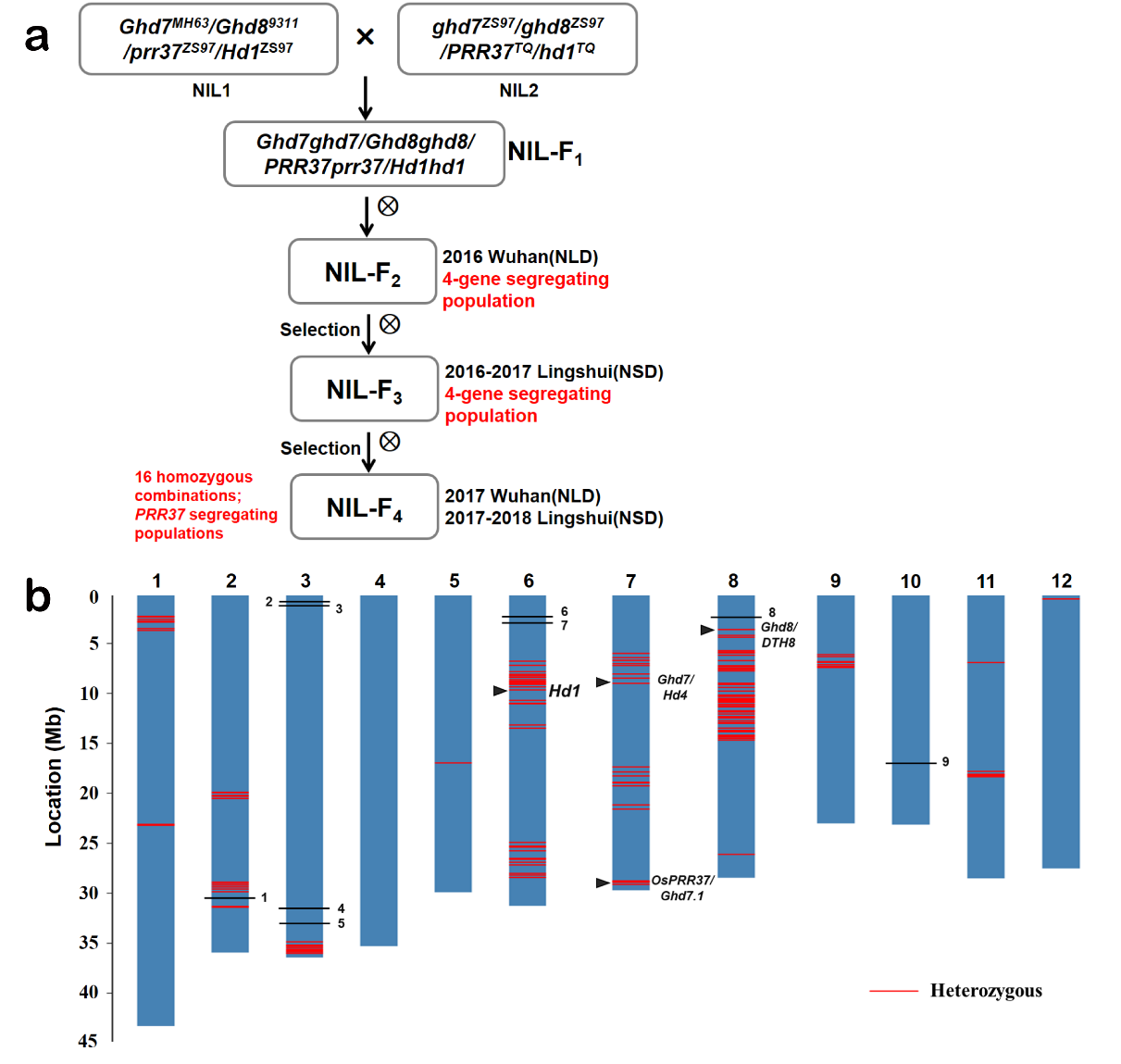


**Figure S1.** Development and genome composition of the rice populations.

**a** Development of the near-isogenic population segregating for 4 heading date genes by crossing NIL1 with NIL2. NIL1 was introgressed with functional *Ghd7^MH63^* and *Ghd8^9311^* in ZS97. NIL2 was introgressed with functional *PRR37^TQ^* and nonfunctional *hd1^TQ^* in ZS97. Thus, the NIL-F_1_ plants contained heterozygous *Ghd7*, *Ghd8*, *PRR37* and *Hd1*. **b** Visualization of the genome composition of the NIL-F_1_ plant obtained by using the Rice6K SNP array. The physical positions of *Ghd7*, *Ghd8*, *PRR37* and *Hd1* are marked by black triangles. Other heading date genes are marked by black lines: 1, *DTH2*; 2, *Ehd4*; 3, *OsMADS50/DTH3*; 4, *Hd6*; 5, *Hd16/EL1*; 6, *Hd17/ELF3*; 7, *Hd3a* and *RFT1*; 8, *Hd18*; 9, *Ehd1*. Red lines indicate heterozygous chromosomal regions based on SNP markers.


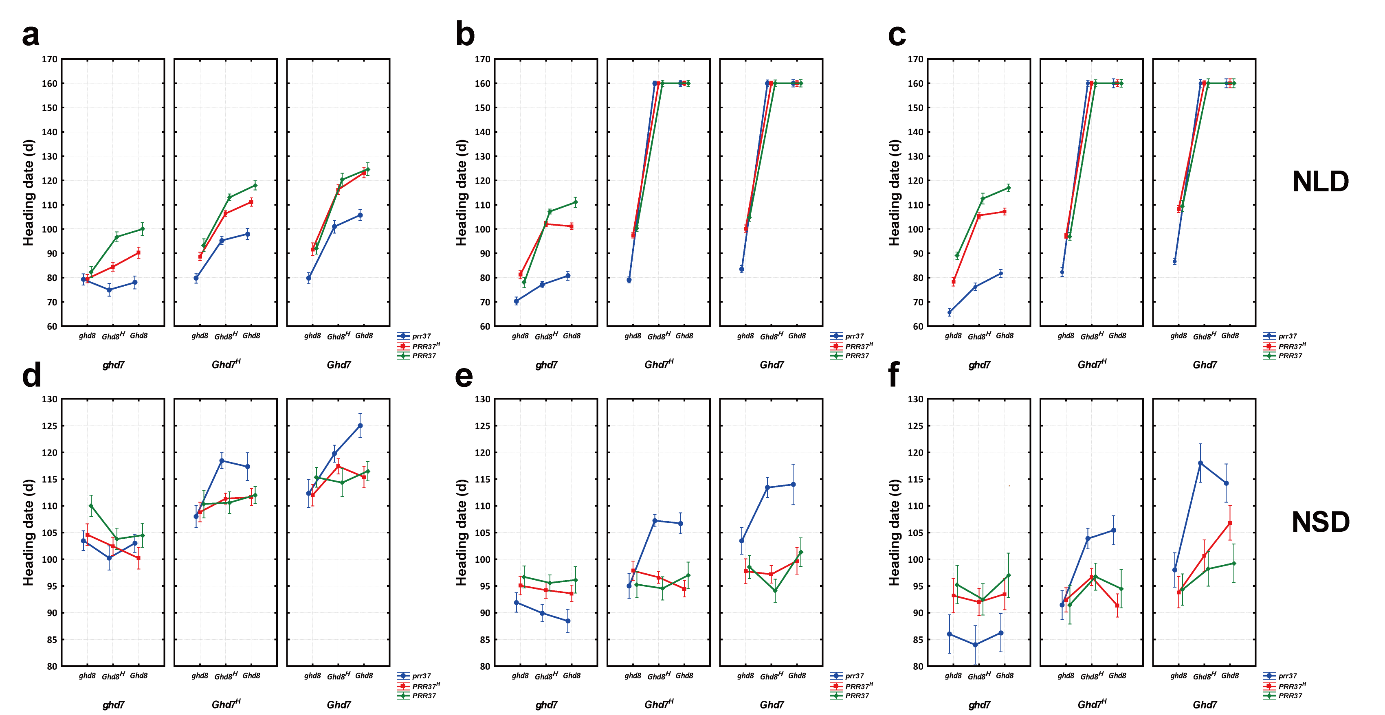


**Figure S2.** Genetic interaction analysis among *Ghd7*, *Ghd8*, *PRR37* and *Hd1* in the 4-gene segregating populations under NLD and NSD conditions.

**a-c** Three-way ANOVA of *Ghd7*, *Ghd8* and *PRR37* in the *Hd1* **a**, *Hd1^H^* **b** and *hd1* **c** backgrounds of NIL-F_2_ under NLD conditions, respectively. **d-f** Three-way ANOVA of *Ghd7*, *Ghd8* and *PRR37* in the *Hd1* **d**, *Hd1^H^* **e** and *hd1* **f** backgrounds of NIL-F_3_ under NSD conditions, respectively. *Ghd7^H^*, *Ghd8^H^* and *PRR37^H^* indicate the heterozygous alleles of *Ghd7*, *Ghd8* and *PRR37*, respectively. Data are represented by LS means. Vertical bars denote 0.95 confidence intervals.


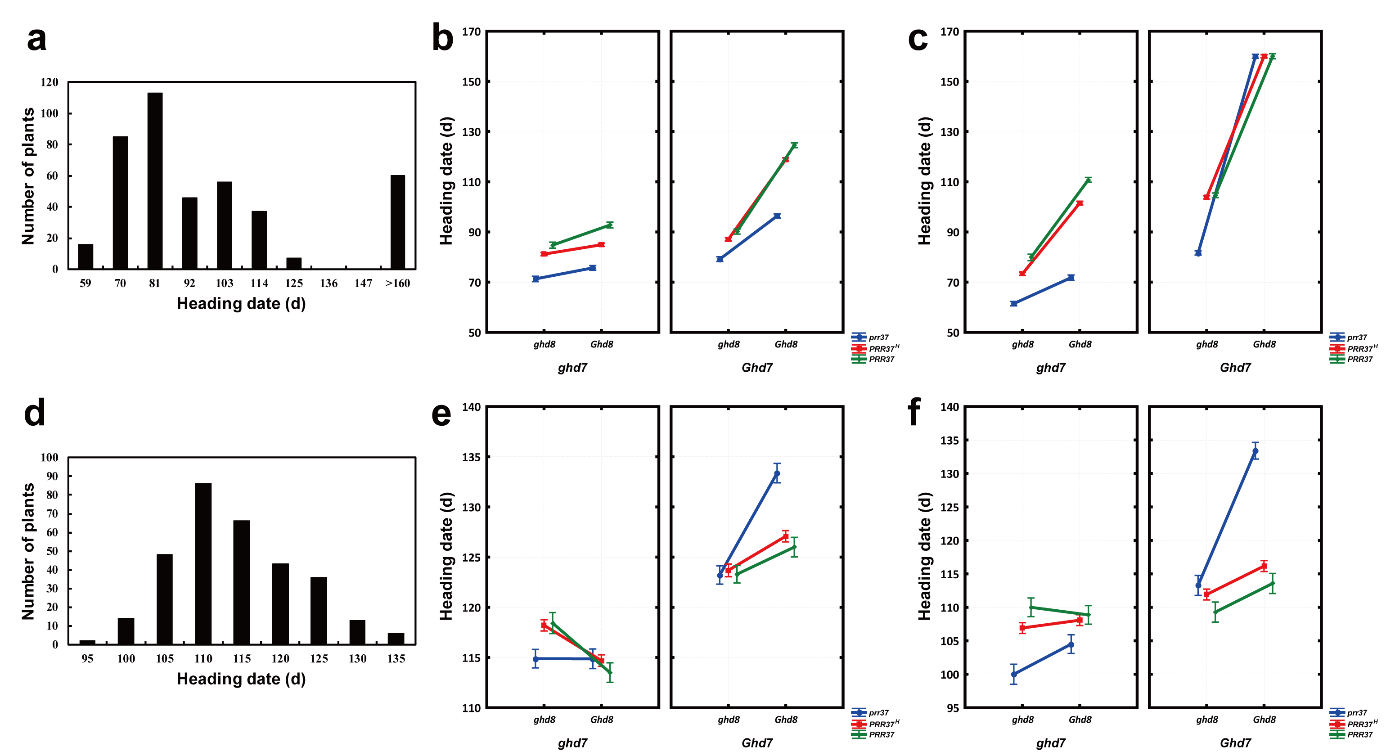


**Figure S3.** Genetic interaction analysis of *Ghd7*, *Ghd8*, *PRR37* and *Hd1* in the merged *PRR37*-segregating populations (NIL-F_4_) under NLD and NSD conditions.

**a** and **d** The distribution of heading date under NLD and NSD conditions, respectively. Factorial ANOVA analysis of *Ghd7*, *Ghd8* and *PRR37* in the *hd1* **b** and *Hd1* **c** backgrounds under NLD conditions, respectively. Factorial ANOVA analysis of *Ghd7*, *Ghd8* and *PRR37* in the *hd1* **e** and *Hd1* **f** backgrounds under NSD conditions, respectively. *PRR37^H^* indicates the heterozygous alleles of *PRR37.* Data are represented by LS means. Vertical bars denote 0.95 confidence intervals.


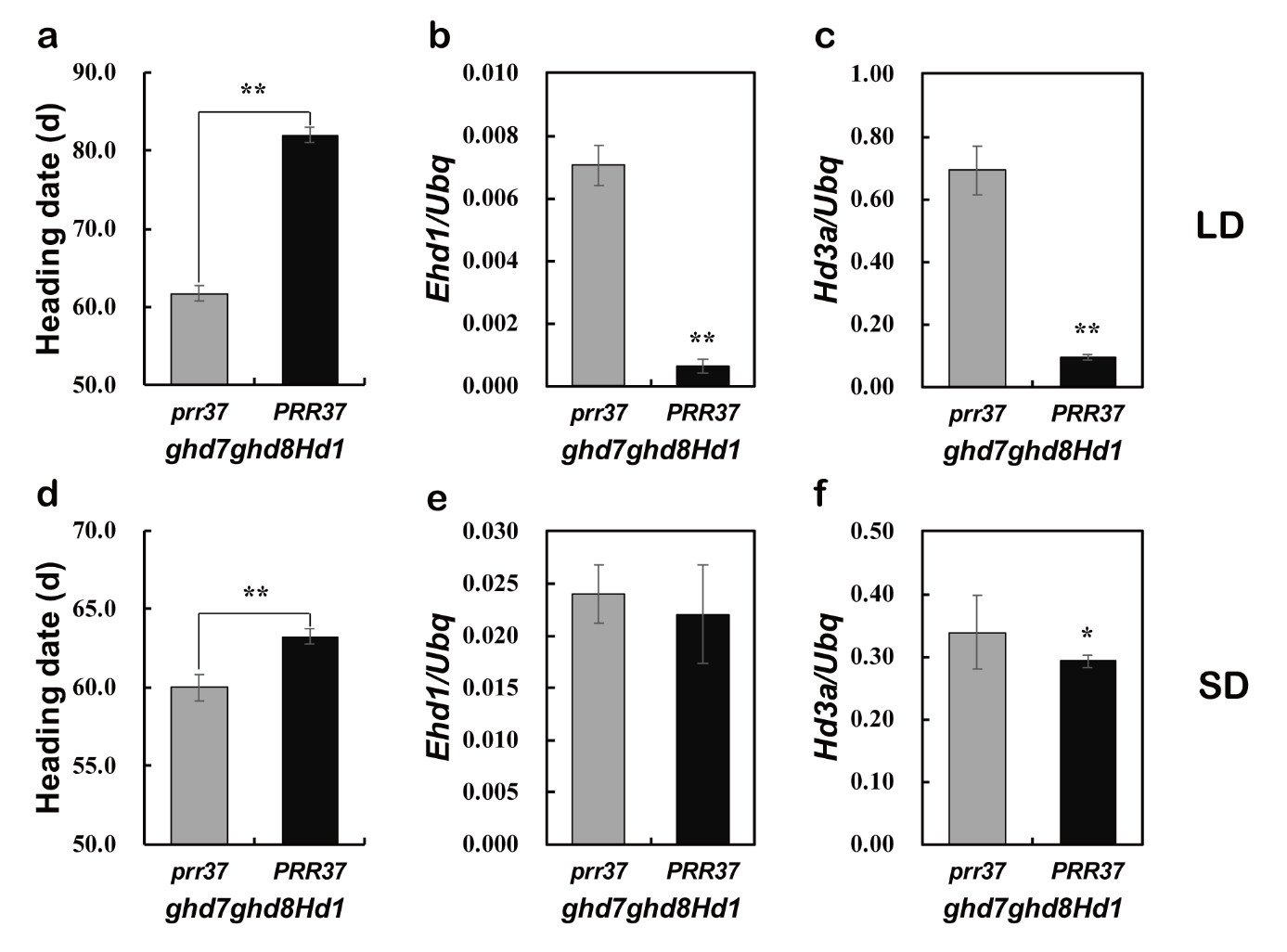


**Figure S4**. *PRR37* delays the heading date in the *ghd7ghd8Hd1* background under both LD and SD conditions.

Heading date **a**, *Ehd1* expression **b** and *Hd3a* expression **c** in *prr37* and *PRR37* lines in the *ghd7ghd8Hd1* background under LD conditions; Heading date **d**, *Ehd1* expression **e** and *Hd3a* expression **f** in *prr37* and *PRR37* in the *ghd7ghd8Hd1* background under SD conditions. * and **, *P*<0.05 and *P*<0.01 based on Student’s *t*-test, respectively.

**Table S1.** Characteristics of four heading date genes and linked markers.

| **Gene** | **Varieties donated alleles** | | **Makers^a^** | **Reference** |
| --- | --- | --- | --- | --- |
|  | **Non-functional** | **Functional** |  |  |
| ***Ghd7*** | ZS97 (Completely absent) | MH63 | MRG4436 | Xue et al. 2008 |
| ***Ghd8*** | ZS97 (1116-bp deletion in 3’ region) | 9311 | Z9M | Yan et al. 2011 |
| ***PRR37*** | ZS97 (Frame shift, 8bp deletion) | Teqing | InDel37 | Yan et al. 2013 |
| ***Hd1*** | Teqing (Frame shift, 4bp deletion) | ZS97 | S56 | Zhang et al. 2017 |

**^a^**MRG4436 is a marker tightly linked to *Ghd7*, and the other three markers are functional markers derived from the corresponding genes.

**Table S2.** The monthly average day length of growing seasons at Wuhan and Lingshui (<https://www.timeanddate.com>).

| **Location** | **Monthly average day length** (h) | | | | | | | | | |
| --- | --- | --- | --- | --- | --- | --- | --- | --- | --- | --- |
|  | Dec | Jan | Feb | Mar | Apr | May | Jun | Jul | Aug | Sep |
| **Wuhan** (30.5°N) |  |  |  |  |  | 13.7 | 14.1 | 13.9 | 13.2 | 12.3 |
| **Lingshui** (18.5°N) | 11.0 | 11.2 | 11.5 | 12.0 | 12.6 |  |  |  |  |  |

**Table S3.** Haplotypes of 10 heading date genes in ZS97.

| **Gene name** | **RAP_LOC** | **RIGW_LOC** | **Haplotype in ZS97** | **Fun.^a^** | **Reference** |
| --- | --- | --- | --- | --- | --- |
| *DTH2* | Os02g0724000 | OsZS_02G0520700 | DTH2-i/Group A | N | Wu et al. 2013 |
| *Ehd4* | Os03g0112700 | OsZS_03G0010800 | Hap_2 | W | Gao et al. 2013 |
| *DTH3/ OsMADS50* | Os03g0122600 | OsZS_03G0020300 | Hap_DJY1**^b^** | F | Lee et al. 2004; Bian et al. 2011 |
| *Hd6* | Os03g0762000 | OsZS_03G0539700 | Hap_Kasalath | F | Takahashi et al. 2001 |
| *Hd16/EL1* | Os03g0793500 | OsZS_03G0567200 | Type 4 | F | Hori et al. 2013; Kwon et al. 2014 |
| *Hd17* | Os06g0142600 | OsZS_06G0037600 | Hap_Koshihikari | W | Matsubara et al. 2012 |
| *RFT1* | Os06g0157500 | OsZS_06G0050900 | Type IIb | N | Zhao et al. 2015 |
| *Hd3a* | Os06g0157700 | OsZS_06G0051000 | Type 3 | F | Takahashi et al. 2009 |
| *Hd18* | Os08g0143300 | OsZS_08G0040400 | Hap_Hayamasari | W | Shibaya et al. 2016 |
| *Ehd1* | Os10g0463400 | OsZS_10G0409200 | Type 6 | F | Takahashi et al. 2009 |

**^a^**Function of allele in ZS97; F, functional; N, nonfunctional; W, weak functional. **^b^**The haplotype of *DTH3/OsMADS50* in ZS97 is consistent with that in DJY1 (Dianjingyou 1). RAP_LOC, LOC number from RAP-DB; RIGW_LOC, LOC number of ZS97 from Rice Information GateWay (<http://rice.hzau.edu.cn/rice/>).

**Table S4.** The genetic interactions in the 4-gene segregating populations under NLD and NSD conditions.

| **Effect** | **DF** | **NLD (NIL-F_2_, n=509)** | |  | **NSD (NIL-F_3_, n=679)** | |
| --- | --- | --- | --- | --- | --- | --- |
|  |  | **F** | ***P*** |  | **F** | ***P*** |
| *Ghd7 by Ghd8* | 4 | 1436 | <1.0E-10 |  | 36.5 | <1.0E-10 |
| *Ghd7 by PRR37* | 4 | 150 | <1.0E-10 |  | 94.8 | <1.0E-10 |
| *Ghd8 by PRR37* | 4 | 9 | 4.8E-07 |  | 27.9 | <1.0E-10 |
| *Ghd7 by Hd1* | 4 | 1078 | <1.0E-10 |  | 10.6 | 2.5E-08 |
| *Ghd8 by Hd1* | 4 | 1296 | <1.0E-10 |  | 8.1 | 2.4E-06 |
| *PRR37 by Hd1* | 4 | 16 | <1.0E-10 |  | 4.6 | 1.1E-03 |
| *Ghd7 by Ghd8 by PRR37* | 8 | 92 | <1.0E-10 |  | 8.1 | 1.8E-10 |
| *Ghd7 by Ghd8 by Hd1* | 8 | 135 | <1.0E-10 |  | 3.7 | 3.2E-04 |
| *Ghd7 by PRR37 by Hd1* | 8 | 61 | <1.0E-10 |  | 7.8 | 6.1E-10 |
| *Ghd8 by PRR37 by Hd1* | 8 | 35 | <1.0E-10 |  | 1.8 | 0.08 |
| *Ghd7 by Ghd8 by PRR37 by Hd1* | 16 | 12 | <1.0E-10 |  | 2.3 | 2.5E-03 |

DF, degree of freedom.

**Table S5.** The genetic interactions among four genes on the basis of the merged *PRR37*-segragating populations (NIL-F_4_)under NLD and NSD conditions.

| **Effect** | **DF** | **NLD (n=420)** | |  | **NSD (n=314)** | |
| --- | --- | --- | --- | --- | --- | --- |
|  |  | **F** | ***P*** |  | **F** | ***P*** |
| *Ghd7 by Ghd8* | 1 | 7097 | <1.0E-10 |  | 340 | <1.0E-10 |
| *Ghd7 by PRR37* | 2 | 83 | <1.0E-10 |  | 233 | <1.0E-10 |
| *Ghd8 by PRR37* | 2 | 42 | <1.0E-10 |  | 129 | <1.0E-10 |
| *Ghd7 by Hd1* | 1 | 5523 | <1.0E-10 |  | 1 | 0.29 |
| *Ghd8 by Hd1* | 1 | 5139 | <1.0E-10 |  | 92 | <1.0E-10 |
| *PRR37 by Hd1* | 2 | 38 | <1.0E-10 |  | 4 | 0.03 |
| *Ghd7 by Ghd8 by PRR37* | 2 | 147 | <1.0E-10 |  | 32 | 3.8E-13 |
| *Ghd7 by Ghd8 by Hd1* | 1 | 571 | <1.0E-10 |  | 0 | 0.82 |
| *Ghd7 by PRR37 by Hd1* | 2 | 265 | <1.0E-10 |  | 76 | <1.0E-10 |
| *Ghd8 by PRR37 by Hd1* | 2 | 84 | <1.0E-10 |  | 11 | 2.7E-05 |
| *Ghd7 by Ghd8 by PRR37 by Hd1* | 2 | 647 | <1.0E-10 |  | 11 | 2.6E-05 |

DF, degree of freedom.

**Table S6.** The heading date and spikelets per panicle of 16 homozygous 4-gene combinations under NLD and NSD conditions.

| **Combination** | **NLD** | |  | **NSD** | |
| --- | --- | --- | --- | --- | --- |
|  | Heading date (d) | Spikelets per panicle |  | Heading date (d) | Spikelets per panicle |
| *ghd7ghd8prr37hd1* | 77.4±1.6 | 172.0±9.8 |  | 113.5±2.0 | 160.8±11.8 |
| *ghd7ghd8PRR37hd1* | 86.7±1.4 | 199.9±7.3 |  | 118.2±2.1 | 157.5±21.5 |
| *ghd7ghd8prr37Hd1* | 60.8±1.8 | 128.2±13.8 |  | 98.7±4.8 | 112.1±7.7 |
| *ghd7ghd8PRR37Hd1* | 77.6±1.8 | 165.8±11.8 |  | 111.3±2.6 | 152.9±8.1 |
| *ghd7Ghd8prr37hd1* | 78.5±1.4 | 174.1±13.0 |  | 114.8±1.8 | 156.2±11.7 |
| *ghd7Ghd8PRR37hd1* | 92.8±0.9 | 174.5±14.5 |  | 113.1±1.3 | 160.9±8.6 |
| *Ghd7ghd8prr37hd1* | 78.9±0.8 | 174.5±8.9 |  | 117.2±0.9 | 152.6±10.6 |
| *Ghd7ghd8PRR37hd1* | 90.1±1.5 | 173.0±7.2 |  | 119.8±1.9 | 165.3±8.7 |
| *ghd7Ghd8prr37Hd1* | 67.1±0.8 | 168.1±9.9 |  | 102.5±3.3 | 117.6±12.1 |
| *ghd7Ghd8PRR37Hd1* | 113.6±2.1 | 136.8±13.7 |  | 107.9±2.6 | 138.6±9.7 |
| *Ghd7ghd8prr37Hd1* | 82.6±1.2 | 184.1±9.8 |  | 109.8±1.5 | 141.7±14.1 |
| *Ghd7ghd8PRR37Hd1* | 111.1±1.0 | 161.4±22.8 |  | 107.8±2.1 | 145.0±11.1 |
| *Ghd7Ghd8prr37hd1* | 97.8±1.2 | 160.7±8.1 |  | 131.8±0.9 | 165.5±13.5 |
| *Ghd7Ghd8PRR37hd1* | 129.2±1.3 | 129.4±12.6 |  | 126.9±0.9 | 159.6±11.8 |
| *Ghd7Ghd8prr37Hd1* | 160^a^ |  |  | 131.3±1.9 | 169.4±9.4 |
| *Ghd7Ghd8PRR37Hd1* | 160^a^ |  |  | 115.1±2.4 | 151.7±8.7 |

**^a^**Non-heading but recorded as 160 days.

**Table S7.** Primers used in this study.

| **Primer name** | **Purpose** | **Gene** | **Forward (5’-3’)** | **Reverse (5’-3’)** |
| --- | --- | --- | --- | --- |
| MRG4436 | genotyping | *Ghd7* | CAAAGGGGGTGTCCTCTATG | GTTGCTCGTCCTACATGTGC |
| Z9M | genotyping | *Ghd8* | GTCGTAGTTTGATCATCACCT | CTTGGTTGTTTGCATTACATG |
| InDel37 | genotyping | *PRR37* | GTGTCCATTAGCCTTAACAGC | CAAGGTTCTAATGGTAGTAGC |
| S56 | genotyping | *Hd1* | GCCAGGAAGTTTGAGAAGAC | CTGCACATCTGATCTCTTGG |
| qEhd1 | qRT-PCR | *Ehd1* | TGGAAATCTCGAAAAACCCG | GCGCTAGCAAAGCTTCGGT |
| qHd3a | qRT-PCR | *Hd3a* | GCTCACTATCATCATCCAGCATG | CCTTGCTCAGCTATTTAATTGCATAA |
| Ubq | qRT-PCR | *Ubiquitin* | AACCAGCTGAGGCCCAAGA | ACGATTGATTTAACCAGTCCATGA |

**References**

Bian X F, Liu X, Zhao Z G et al (2011) Heading date gene, *dth3* controlled late flowering in O. Glaberrima Steud. by down-regulating Ehd1. Plant Cell Reports 30**:** 2243-2254.

Gao H, Zheng X M, Fei G et al (2013) *Ehd4* encodes a novel and *Oryza-genus*-specific regulator of photoperiodic flowering in rice. PLoS Genet 9**:** e1003281.

Hori K, Ogiso-Tanaka E, Matsubara K et al (2013) *Hd16*, a gene for casein kinase I, is involved in the control of rice flowering time by modulating the day-length response. Plant J 76**:** 36-46.

Kojima S, Takahashi Y, Kobayashi Y et al (2002) *Hd3a*, a rice ortholog of the Arabidopsis *FT* gene, promotes transition to flowering downstream of *Hd1* under short-day conditions. Plant Cell Physiol 43**:** 1096-1105.

Kwon C T, Yoo S C, Koo B H et al (2014) Natural variation in *Early flowering 1* contributes to early flowering in japonica rice under long days. Plant Cell Environ 37**:** 101-112.

Lee S, Kim J, Han J J et al (2004) Functional analyses of the flowering time gene *OsMADS50*, the putative *SUPPRESSOR OF OVEREXPRESSION OF CO 1*/*AGAMOUS-LIKE 20 (SOC1/AGL20)* ortholog in rice. Plant J 38**:** 754-764.

Matsubara K, Ogiso-Tanaka E, Hori K et al (2012) Natural Variation in *Hd17*, a Homolog of Arabidopsis *ELF3* That is Involved in Rice Photoperiodic Flowering. Plant and Cell Physiology 53**:** 709-716.

Shibaya T, Hori K, Ogiso-Tanaka E et al (2016) *Hd18*, Encoding Histone Acetylase Related to Arabidopsis *FLOWERING LOCUS D*, is Involved in the Control of Flowering Time in Rice. Plant Cell Physiol 57**:** 1828-1838.

Takahashi Y, Shomura A, Sasaki T et al (2001) *Hd6*, a rice quantitative trait locus involved in photoperiod sensitivity, encodes the alpha subunit of protein kinase CK2. Proc Natl Acad Sci U S A 98**:** 7922-7927.

Takahashi Y, Teshima K M, Yokoi S et al (2009) Variations in *Hd1* proteins, *Hd3a* promoters, and Ehd1 expression levels contribute to diversity of flowering time in cultivated rice. Proc Natl Acad Sci U S A 106**:** 4555-4560.

Wu W, Zheng X M, Lu G et al (2013) Association of functional nucleotide polymorphisms at *DTH2* with the northward expansion of rice cultivation in Asia. Proc Natl Acad Sci USA 110**:** 2775-2780.

Xue W, Xing Y, Weng X et al (2008) Natural variation in *Ghd7* is an important regulator of heading date and yield potential in rice. Nat Genet 40**:** 761-767.

Yan W, Liu H, Zhou X et al (2013) Natural variation in *Ghd7.1* plays an important role in grain yield and adaptation in rice. Cell Res 23**:** 969-971.

Yan W H, Wang P, Chen H X et al (2011) A major QTL, *Ghd8*, plays pleiotropic roles in regulating grain productivity, plant height, and heading date in rice. Mol Plant 4**:** 319-330.

Zhang Z, Hu W, Shen G et al (2017) Alternative functions of Hd1 in repressing or promoting heading are determined by Ghd7 status under long-day conditions. Sci Rep 7**:** 5388.

Zhao J, Chen H, Ren D et al (2015) Genetic interactions between diverged alleles of *Early heading date 1* (*Ehd1*) and *Heading date 3a* (*Hd3a*)/ *RICE FLOWERING LOCUS T1* (*RFT1*) control differential heading and contribute to regional adaptation in rice (*Oryza sativa*). New Phytol 208**:** 936-948.
